# Efficient Single-Shot Multi-Object Tracking for Vehicles in Traffic Scenarios

**DOI:** 10.3390/s21196358

**Published:** 2021-09-23

**Authors:** Youngkeun Lee, Sang-ha Lee, Jisang Yoo, Soonchul Kwon

**Affiliations:** 1 Department of Electronic Engineering, Kwangwoon University, Seoul 01897, Korea; yklee1308@kw.ac.kr (Y.L.); kcv456@kw.ac.kr (S.-h.L.); jsyoo@kw.ac.kr (J.Y.); 2 Graduate School of Smart Convergence, Kwangwoon University, Seoul 01897, Korea

**Keywords:** multi-object tracking, object detection, single-shot, traffic scenario, vehicle tracking

## Abstract

Multi-object tracking is a significant field in computer vision since it provides essential information for video surveillance and analysis. Several different deep learning-based approaches have been developed to improve the performance of multi-object tracking by applying the most accurate and efficient combinations of object detection models and appearance embedding extraction models. However, two-stage methods show a low inference speed since the embedding extraction can only be performed at the end of the object detection. To alleviate this problem, single-shot methods, which simultaneously perform object detection and embedding extraction, have been developed and have drastically improved the inference speed. However, there is a trade-off between accuracy and efficiency. Therefore, this study proposes an enhanced single-shot multi-object tracking system that displays improved accuracy while maintaining a high inference speed. With a strong feature extraction and fusion, the object detection of our model achieves an AP score of 69.93% on the UA-DETRAC dataset and outperforms previous state-of-the-art methods, such as FairMOT and JDE. Based on the improved object detection performance, our multi-object tracking system achieves a MOTA score of 68.5% and a PR-MOTA score of 24.5% on the same dataset, also surpassing the previous state-of-the-art trackers.

## 1. Introduction

In recent years, numerous studies have been conducted to implement multi-object tracking (MOT), which is a process that predicts trajectories of multiple objects detected across a sequence of video frames [1,2,3,4,5,6,7]. Among all the approaches, deep learning-based methods have shown remarkable improvements and have enabled several applications, such as video surveillance and analysis [8], human recognition and communication [9], and autonomous driving [10].

Deep learning-based MOT systems in early works have been composed of two separate and sequential processes: object detection and embedding extraction [2,3]. The two processes operate in a sequential manner, in which the bounding boxes of objects are first predicted by an object detection model, and then the appearance embeddings are extracted by an embedding extraction model to determine whether the objects are matched to existing trajectories. Object detection and embedding extraction are independent of each other; thus, they have been developed respectively, and the main focus has been to finely combine these two processes. Despite the high accuracy of two-stage methods, their inference speed is severely limited, which makes their real-time applications infeasible.

To address the aforementioned limitation, single-shot approaches, which apply a parallel structure to object detection and embedding extraction, have been developed [4,5,6,7]. Single-shot methods simultaneously predict the locations and extract the appearance embeddings of objects in separate branches. Therefore, the time gap between the two processes decreases, and the inference speed notably improves. Furthermore, most single-shot methods enhance the inference speed to the point that it can run in real-time by using a lightweight backbone network. However, their accuracy suffers from a trade-off from a high inference speed because it is difficult to extract rich features with a small network size.

In this study, we propose a fast single-shot MOT system that achieves high accuracy. By using an optimized backbone network and an efficient feature fusion, our proposed method shows higher MOT accuracy without significantly reducing the efficiency. In particular, our system exhibits an outstanding performance on vehicle tracking tasks, showing improvements on the experimental results on the public UA-DETRAC dataset [11].

## 2. Related Works

Multi-object tracking has become highly challenging in the last decade, requiring complex multi-stage algorithms and high-end hardware performance, such as computing memory and speed [1]. Meanwhile, deep learning and processing units have made rapid advancements and have actualized MOT to the level of real-world applications [2,3,4,5,6,7]. In particular, previous studies on a wide range of fields in deep learning, such as image classification [12,13,14,15,16] and object detection [17,18,19,20,21,22,23,24,25], have inspired and guided researchers to devise groundbreaking solutions for MOT.

Image classification is one of the earliest tasks in computer vision that uses deep learning. LeNet-5 [12] marked a watershed in the history of deep learning in computer vision by proposing a gradient-based learning of a convolutional neural network (CNN), which successfully enhanced the identification of handwritten numbers. Successive studies, such as AlexNet [13], VGGNet [14], GoogLeNet [15], and ResNet [16], improved the performance of image classification with thorough analysis and various modifications to the structures of CNNs, training strategies, and other details.

With the development of image classification, several studies have been conducted on object detection, which is a process of predicting the locations and classes of objects in an image or sequence of video frames. Object detection using deep learning can be classified into two-stage [17,18,19,20] and one-stage methods [21,22,23,24,25]. Two-stage detectors, such as the R-CNN family [17,18,19,20], operate sequentially in two steps, in which they first make region proposals and then classify each one into a class. In contrast, one-stage detectors, such as the YOLO family [21,22,23], SSD [24], and RetinaNet [25], simultaneously carry out localization and classification.

Based on the foundational studies on object detection, several studies have been proposed for matching detected objects with IDs across frames to implement MOT. The ID-matching procedures can be classified into non-deep learning [1] and deep learning-based methods [2,3,4,5,6,7]. Non-deep learning methods perform data association with only the locations of the objects. SORT [1] is a widely used non-deep learning data association algorithm, which links the current object locations with the previous object locations by applying the Kalman filter [26]. In particular, SORT associates objects based on the Intersection over Union (IoU) and facilitates matching using the Hungarian algorithm [27]. Non-deep learning methods can be utilized for any detector, which is a great advantage; however, they have a great weakness for occlusions.

To solve the difficulty of non-deep learning methods in tracking overlapping objects, few approaches for matching IDs based on deep learning have been suggested. Compared to non-deep learning methods, deep learning-based methods use not only locations but also appearance embeddings, which are embedding vectors that represent the appearance information of objects, such as the shape and color. Early studies, such as POI [2] and Deep SORT [3], successfully developed a deep learning-based model of appearance embeddings as a subsequent object detection process. Both POI and Deep SORT first predict the bounding boxes of objects and then forward-pass the cropped regions to extract the appearance embeddings. POI utilizes the GoogleNet [15]-based network for embedding extraction and applies the cosine distances to the integrated predicted motions and visual information for data association. Deep SORT uses the squared Mahalanobis distances for motions and the cosine distances for appearance embeddings, respectively. Despite the increase in MOT accuracy, two-stage trackers have issues with inference speed because they perform object detection and embedding extraction sequentially. As a solution, single-shot trackers that concurrently perform the two processes have been proposed. JDE [4] first extracts and fuses the features by using the backbone network of YOLOv3 [23], and then predicts the locations and classes of objects in each prediction head with the extraction of appearance embeddings. FairMOT [5] utilizes DLA [28] for a feature fusion and employs a heatmap head to predict the centers of the objects. Both JDE and FairMOT achieved a speed of 30 FPS, enabling a real-time operation of deep learning-based MOT systems.

Vehicle tracking is one of the most important tasks in MOT owing to its extensive applications, such as traffic monitoring, analysis, and control [8]. The vehicle-tracking task has several challenges, including tracking objects in various sizes and views, in different environments of illuminations, and with heavy occlusions. Several studies have suggested robust MOT systems as a solution for tracking vehicles in traffic scenarios recorded by an unmanned aerial vehicle or a self-driving car. Early approaches have used non-deep learning methods that combine detectors, such as DPM [29], ACF [30], R-CNN [17], and CompACT [31], with data association algorithms, such as GOG [32], CEM [33], DCT [34], IHTLS [35], H${}^{2}$T [36], and CMOT [37]. Bochinski et al. [38] proposed an integration of Mask R-CNN [20] and V-IOU, which performed well on the UA-DETRAC dataset [11] and achieved a PR-MOTA score of 30.7%. Meanwhile, considerable approaches that apply deep learning-based methods to vehicle tracking have also been made. Track R-CNN [6] simultaneously performs MOT and segmentation with a single CNN by training its network with the pixel-level annotated KITTI [39] and MOT17 [40] benchmarks. DMM-Net [7] predicts motion parameters of anchor tubes and directly associates the tracklets based on the IoU. By using the tracklet association, DMM-Net tracked vehicles at a highly enhanced speed (123.3 FPS) and achieved a PR-MOTA score of 12.8% on the UA-DETRAC dataset. Single-shot vehicle trackers have made notable developments in speed; however, their accuracy has not been fully focused on in prior works. Therefore, considering future applications of vehicle tracking, it is important to improve the accuracy and not only the efficiency.

## 3. Proposed Method

In this section, we propose a backbone network and a method for output-feature fusion for accurate and efficient single-shot MOT. Among many MOT models, we adopt FairMOT [5] as a baseline model since it operates at a high inference speed by decreasing the time gap between object detection and embedding extraction. For rich feature extraction and fusion, we replace the original backbone network with a strong yet simple network that performs optimized cross-scale connections. In addition, we show how we can effectively integrate the multi-scale features output from the backbone network for subsequent operations.

**Figure 1:**
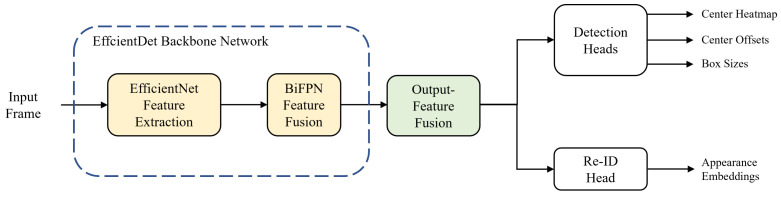
Overall flowchart of our single-shot multi-object tracking (MOT) system.

The overall flowchart of our single-shot MOT system is shown in Figure 1. The MOT system begins by forward-passing an input frame into the backbone network. We use EfficientDet [41] as a backbone network; it consists of two stages: feature extraction and fusion. In the first stage, EfficientNet [42] performs feature extraction and produces three multi-scale feature maps. In the second stage, the features go through the process of feature fusion via the bi-directional feature pyramid network (BiFPN) [41] layers and five multi-scale feature maps are taken out from the backbone network. Before being transferred to prediction heads, the output features are fused into one to match the feature dimensions. In Section 3.1 and Section 3.2, we describe the backbone network and output-feature fusion of the proposed method, respectively. For predictions of the center heatmap, center offsets, and box sizes of the objects, the integrated feature is transferred to the detection head. Likewise, the same procedure is carried out for the re-ID head to extract appearance embeddings from the feature map. Finally, online association is applied to the output results from the prediction heads to match the IDs of objects in the current and previous frames.

### 3.1. Backbone Network Architecture

A backbone network plays a significant role in the overall MOT system, in that it generates features that are essential for further steps. The performance of a MOT system varies greatly, depending on how the backbone network extracts and aggregates high-quality features with its own method. In our proposed method, we adopt EfficientDet [41] as a backbone network to improve the performance of MOT by increasing the accuracy while minimizing the loss of efficiency. We present two reasons for using EfficientDet. EfficientNet [42], which is the backbone network of EfficientDet, shows outstanding performance for a small number of parameters, and achieves high accuracy and a fast inference speed. The second reason is that BiFPN [41], which is the fusion network, increases the average precision (AP) whilst having a lower computational cost, as compared to others [43,44].

**Figure 2:**
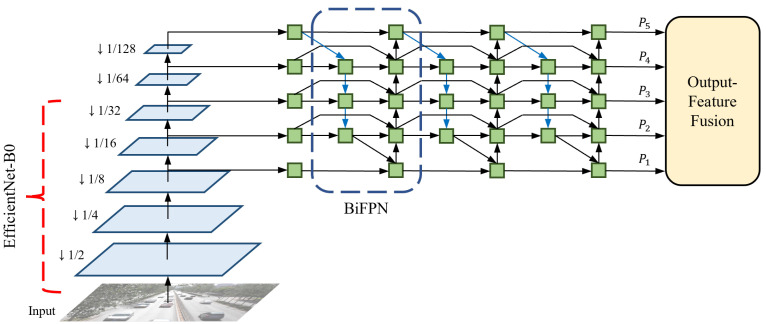
Backbone network architecture of our single-shot MOT system.

EfficientDet offers eight different optimized models based on thorough observations by considering depth (number of layers), width (number of channels), and input resolution. Among them, we utilize EfficientDet-D0, which is the most lightweight model that repeats the BiFPN layers three times, uses 64 channels, and takes an input resolution of 512 × 512. EfficientDet-D0 is selected to minimize the decrease in the inference speed. This is because scaling up the three factors reduces the efficiency to the extent where it differs significantly from the baseline [5]. The backbone network architecture of the proposed method is shown in Figure 2. An input frame is first forward-passed into the backbone network, and EfficientNet produces three multi-scale features with resolutions of 8, 16, and 32 times lower than the input size, respectively. In this process, EfficientNet effectively decreases the parameters and FLOPs by using the mobile inverted bottleneck convolution (MBConv) [45,46]. As a pair, EfficientNet-B0 is applied to the feature extractor. The original EfficientNet-B0 operates 18 convolutions of different kernel sizes, including two standard convolutions and 16 MBConvs, and uses a fully-connected layer for classification. However, because EfficientNet-B0 only performs feature extraction without additional processes of classification in EfficientDet-D0, the last 1 × 1 convolutional layer and the following fully-connected layer are removed from the original model. The structure and specifications of the modified EfficientNet-B0 are listed in Table 1.

The following step details how to fuse the multi-scale features with BiFPN. Before sending the three multi-scale features to the BiFPN layers, a 1 × 1 convolution is performed on each feature map to set the number of channels to 64 and two additional lower-resolution features are extracted by max pooling. Therefore, a total of five multi-scale features, with the same number of channels, are transferred to the BiFPN layers. BiFPN effectively fuses features by applying several techniques to PANet [47], which utilizes both top-down and bottom-up pathways. The modifications for optimization are as follows: (1) Cut off nodes that have negligible influence on the quality of feature maps. (2) Add extra skip connections. (3) Repeat the entire BiFPN layer multiple times. By leveraging the optimized network, the output features {$P_{1},\;P_{2},\;P_{3},\;P_{4},\;P_{5}$} of the backbone network become highly robust to scale variations.

Input resolution greatly affects the development of a CNN. In particular, it becomes significantly important in tasks that require rich representations of features for small objects, such as object detection and MOT. In general, a larger input resolution is accompanied by a larger network size and results in higher accuracy. However, the increase in both factors critically harms the inference speed by drastically increasing the computations. Therefore, it is necessary to scrutinize the performance of the CNN with different input sizes and different numbers of network parameters to find the optimal combination that optimizes the balance between accuracy and efficiency. In Tan et al. [41], EfficientDet-D0 is designed to operate with an input resolution of 512 × 512. In our method, we intentionally set an input size of 1024 × 512 (width × height). This idea is based on the observation that simply increasing the input resolution of a lightweight network benefits the efficiency more than using a heavyweight network of large resolution, while both show similar performances for the accuracy. An aspect ratio of the input is also significant for the performance of the CNN. This is because a wide discrepancy in the aspect ratio between the input and frame leads to a heavy loss of information when the frame is resized. Considering the frame size of videos in real-world applications, the aspect ratio is set to 2:1 in our proposed method. The input resolution of the feature maps for each operation is shown in Table 1.

### 3.2. Output-Feature Fusion

Output-feature fusion is a vital process for integrating multi-scale features output from a backbone network into a single feature map for subsequent operations. Based on previous studies that utilize heatmaps for predictions [48,49,50,51], our single-shot MOT system performs object detection and embedding extraction on a single high-resolution feature map. Therefore, it is crucially important for our network to generate a single feature that potentially comprises rich representations.

**Figure 3:**
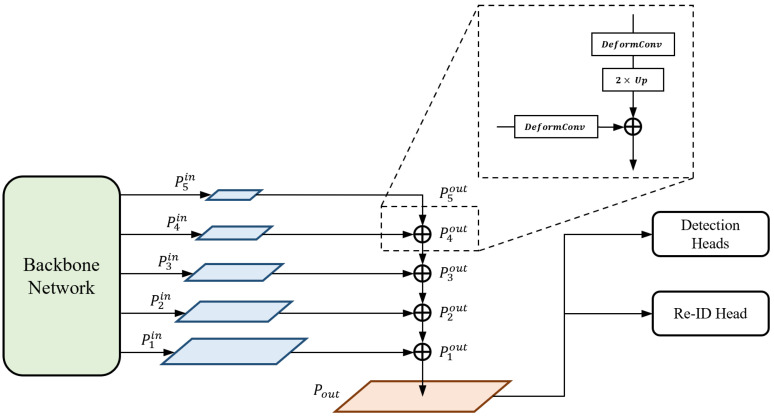
Output-feature fusion network architecture of our proposed method.

The output-feature fusion network architecture of the proposed method is illustrated in Figure 3. We append the structure of the feature pyramid network (FPN) [43] to the end of the backbone network for fusion. FPN serially merges multi-scale features from high to low levels to produce semantically strong feature maps. The features from the top-down pathway and lateral connections are fused using Equation (1):

(1) $$\begin{array}{c} P_{5}^{out}=P_{5}^{in}\\ P_{4}^{out}=Up\left(DeformConv\left(P_{5}^{out}\right)\right)+DeformConv\left(P_{4}^{in}\right)\\ \cdots \\ P_{1}^{out}=Up\left(DeformConv\left(P_{2}^{out}\right)\right)+DeformConv\left(P_{1}^{in}\right) \end{array}$$

where {$P_{1}^{in},\;P_{2}^{in},\;P_{3}^{in},\;P_{4}^{in},\;P_{5}^{in}$} are the multi-scale input features transferred from the backbone network, {$P_{1}^{out},P_{2}^{out},P_{3}^{out},P_{4}^{out},P_{5}^{out}$} are the output features, DeformConv is the deformable convolutional operation, and Up is the up-scaling operation. At each level, a deformable convolution [52] is first performed on both features, which are from the higher level and lateral connection, respectively. Inspired by [5], we use the deformable convolution for every branch in order to adaptively decide the receptive field by applying a learnable offset, which varies depending on the scales of an object, to the grid point of kernels. Subsequently, a lower-resolution feature is up-scaled by a factor of two by the transposed convolution; thus, it is resized to the same resolution as the other. We adopt the concept of the depth-wise convolution [53] here to reduce the number of parameters and computations. Finally, the feature maps of the same spatial size are fused by element-wise summation and the output is sent to the lower level in order to repeat the entire fusion block.

To generate a final high-resolution feature map that is transferred to prediction heads, we up-scale the feature $P_{1}^{out}$ of the highest resolution among the feature maps from FPN. The up-scaling process is identical to the combination of the operations DeformConv and Up, which are used in the top-down pathway of FPN. That is, the feature $P_{1}^{out}$ is up-scaled by Equation (2):

(2) $$P_{out}=Up\left(DeformConv\left(P_{1}^{out}\right)\right)$$

where $P_{out}$ is the final high-resolution feature map. As a result, given an input image $I\in R^{H\times W\times 3}$, where *H* and *W* are the height and width, respectively, the output-feature fusion network outputs the single high-resolution feature map $P_{out}\in R^{\frac{H}{4}\times \frac{W}{4}\times 64}$, which is utilized for subsequent predictions.

### 3.3. Prediction Heads

A prediction head is a significant component in the single-shot MOT system because it determines the specific tasks that the system will perform. In general, prediction heads are appended to the end of a backbone network and receive the extracted features needed to carry out various tasks, such as localization and classification of bounding boxes. In our proposed method, the prediction heads are attached to the end of the output-feature fusion network and take the input of a single high-resolution feature map, whose resolution is four times lower than the size of an input image. Based on a feature with rich representations, we utilize three prediction heads for object detection and one re-ID head for embedding extraction. Here, the three prediction heads for object detection are composed of center-heatmap, center-offset, and box-size heads proposed in CenterNet [50].

In front of each prediction head, a 3 × 3 convolution with 256 channels and a 1 × 1 convolution are performed to make the feature map applicable to the assigned tasks. Specifically, the target of the center-heatmap head has only 1 channel of the heatmap because our single-shot MOT system works for a single class. In the center-offset head, the number of target channels is set to two (for horizontal and vertical offsets), while that in the box-size head is set to four (for top, left, bottom, and right edges of a box). The re-ID head utilizes a target that has 128 channels, which comprise an appearance embedding extracted from an object center.

The center-heatmap head is used to predict the locations of the centers of the objects and aims to estimate the probability of containing the object center at each location of the target heatmap $\hat{Y}\in {\left[0,\;1\right]}^{\frac{H}{4}\times \frac{W}{4}}$. The probability is ideally one at the object center and rapidly decreases as the distance between the predicted location and object center increases. In the training stage, the ground-truth heatmap $Y\in {\left[0,\;1\right]}^{\frac{H}{4}\times \frac{W}{4}}$ is set to a 2D Gaussian mixture, where each Gaussian distribution corresponds to a single object. Given a ground-truth object center $c^{\left(k\right)}\in R^{2}$ in an input image *I*, the location is first converted into the down-scaled object center ${\tilde{c}}^{\left(k\right)}=\left[\frac{c^{\left(k\right)}}{4}\right]$, and then the 2D Gaussian distribution $Y^{\left(k\right)}\in {\left[0,\;1\right]}^{\frac{H}{4}\times \frac{W}{4}}$ is produced by Equation (3):

(3) $$Y^{\left(k\right)}=\exp\left(-\frac{{\left(x-{\tilde{c}}_{x}^{\left(k\right)}\right)}^{2}+{\left(y-{\tilde{c}}_{y}^{\left(k\right)}\right)}^{2}}{2\sigma_{c}^{2}}\right)$$

where $\sigma_{c}$ is the standard deviation, which varies depending on the size of an object. To generate the final ground-truth heatmap *Y*, all Gaussian distributions are merged by element-wise maximum, as shown in Equation (4):

(4) $$Y_{ij}=\max\left(Y_{ij}^{\left(1\right)},Y_{ij}^{\left(2\right)},\cdots ,Y_{ij}^{\left(N\right)}\right)$$

where *N* is the number of objects in the input image. We denote the loss function $L_{heatmap}$ is the focal loss [25] for penalty-reduced pixel-wise logistic regression [49], as shown in Equation (5):

(5) $$L_{heatmap}=-\frac{1}{N}\sum_{i=1}^{H}\sum_{j=1}^{W}\left\{\begin{array}{cc} {\left(1-{\hat{Y}}_{ij}\right)}^{\alpha }\log\left({\hat{Y}}_{ij}\right) & \mathrm{if}\;Y_{ij}=1\\ {\left(1-Y_{ij}\right)}^{\beta }{\left({\hat{Y}}_{ij}\right)}^{\alpha }\log\left(1-{\hat{Y}}_{ij}\right) & \mathrm{otherwise} \end{array}\right.$$

where α and β are the hyperparameters of the modulating factors in the focal loss. In our proposed method, we set α=2 and β=4, adopting the values used in CornerNet [49].

The center-offset head is employed to restore the information of accurate object locations that are lost because of the down-scaling process. The objective of this prediction head is to precisely adjust the positions of objects by applying offsets, which are horizontal and vertical shifts, to the down-scaled object centers. From the target offsets $\hat{O}\in {\left[0,\;1\right]}^{\frac{H}{4}\times \frac{W}{4}\times 2}$, we first sample the offsets ${\hat{o}}^{\left(k\right)}\in {\left[0,\;1\right]}^{2}$ only at the down-scaled object center ${\tilde{c}}^{\left(k\right)}$. Subsequently, the Manhattan distance between the sampled offsets and the ground-truth offsets, which are pre-determined by $o^{\left(k\right)}=\frac{c^{\left(k\right)}}{4}-{\tilde{c}}^{\left(k\right)}$, is computed for regression. We denote the loss function $L_{offset}$ is the L1 loss, as defined by Equation (6):

(6) $$L_{offset}=\frac{1}{N}\sum_{k=1}^{N}\left|{\hat{o}}^{\left(k\right)}-o^{\left(k\right)}\right|$$

The box-size head is utilized for predicting the object sizes by estimating the top, left, bottom, and right edges of the bounding boxes. In our proposed method, the regression of the four edges is used instead of the regression of the height and width for more accurate localization. The training process of the box-size head is similar to that of the center-offset head. Given a ground-truth bounding box $b^{\left(k\right)}=\left(c_{x}^{\left(k\right)},c_{y}^{\left(k\right)},h^{\left(k\right)},w^{\left(k\right)}\right)$, where $h^{\left(k\right)}$ and $w^{\left(k\right)}$ are the height and width, respectively, the ground-truth size is computed as $s^{\left(k\right)}=\left(c_{x}^{\left(k\right)}-\frac{w^{\left(k\right)}}{2},\;c_{y}^{\left(k\right)}-\frac{h^{\left(k\right)}}{2},\;c_{x}^{\left(k\right)}+\frac{w^{\left(k\right)}}{2},\;c_{y}^{\left(k\right)}+\frac{h^{\left(k\right)}}{2}\right)$. Afterward, we compute the Manhattan distance between the ground-truth size and the sampled size ${\hat{s}}^{\left(k\right)}\in R^{4}$ from the target size $\hat{S}\in R^{\frac{H}{4}\times \frac{W}{4}\times 4}$ for the regression. We denote the loss function $L_{size}$ is the L1 loss, as shown in Equation (7):

(7) $$L_{size}=\frac{1}{N}\sum_{k=1}^{N}\left|{\hat{s}}^{\left(k\right)}-s^{\left(k\right)}\right|$$

The re-ID head is used to encode the appearance information of objects, such as shape, color, and view, into the appearance embedding vectors for object re-identification. This head aims to make the embedding vectors of the same-ID objects close to each other and those of the different-ID objects far from each other. In the training stage, we consider all object IDs in the training set as classes. We also append a fully-connected layer that has the same number of output channels of the classes to the re-ID head in order to treat the task as a classification task. From the target of the appearance embedding $\hat{E}\in R^{\frac{H}{4}\times \frac{W}{4}\times 128}$, the embedding vector ${\hat{e}}^{\left(k\right)}\in R^{128}$ is first extracted from the down-scaled object center ${\tilde{c}}^{\left(k\right)}$. Afterward, the class-probability vector $p^{\left(k\right)}\in {\left[0,\;1\right]}^{M}$, where *M* is the number of object IDs in the training set, is generated by the dense layer and softmax operation. Subsequently, the cross entropy between the class-probability vector and the ground-truth one-hot encoded vector $q^{\left(k\right)}\in {\left\{0,\;1\right\}}^{M}$ is computed for regression. We denote the loss function $L_{id}$ is the cross-entropy loss, as defined by Equation (8):

(8) $$L_{id}=-\frac{1}{N}\sum_{k=1}^{N}\sum_{i=1}^{M}q_{i}^{\left(k\right)}\log\left(p_{i}^{\left(k\right)}\right)$$

It is necessary to train multiple prediction heads to optimize our overall single-shot MOT system; thus, we approach this work as a multi-task learning problem. Specifically, the automatic balancing of multiple losses using uncertainty [54] is applied to the multi-task learning of object detection and re-identification. We first define the loss function of detection $L_{det}$ by the weighted summation of losses, as shown in Equation (9):

(9) $$L_{det}=L_{heatmap}+\lambda_{offset}L_{offset}+\lambda_{size}L_{size}$$

where $\lambda_{offset}$ and $\lambda_{size}$ are the loss-weight constants. We set $\lambda_{offset}=1$ and $\lambda_{size}=0.1$, adopting the values used in CenterNet [50]. Finally, the total loss function $L_{total}$ is calculated from Equation (10):

(10) $$L_{total}=\frac{1}{2}\left(\frac{1}{\exp(w_{det})}L_{det}+\frac{1}{\exp(w_{id})}L_{id}+w_{det}+w_{id}\right)$$

where $w_{det}$ and $w_{id}$ are the learnable loss weights of the automatic multi-loss balancing.

### 3.4. Online Association

**Figure 4:**
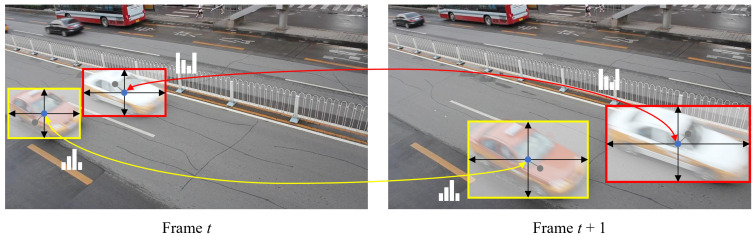
Visualization of online association. The frames are selected from the sequence MVI-40181 in the UA-DETRAC-train set, with a time interval of approximately 10 frames for better visualization.

Online association is a crucial process in MOT because the detected objects are interpreted as tracklets by the online association algorithm. The association algorithm aims to accurately track objects by maximizing the leverage of the appearance embeddings, with aid from the predicted object locations. The visualization of online association is shown in Figure 4. In the first frame of a sequence, tracklets are initially activated by the detected objects. Subsequently, we associate the activated tracklets with the newly detected objects in the next frame based on the cosine distances between the embedding vectors. The costs are computed for every combination of the tracklets and objects, whose confidence score is higher than a threshold; the pairs are subsequently matched by the Hungarian algorithm [27]. Furthermore, we compute the squared Mahalanobis distances between the object centers and state distributions, which are predicted by the Kalman filter [26], to avoid matching the distant different-ID objects with similar appearances.

For efficient tracklet management, we utilize a tracklet pool, which is a set of categorized tracklets. Here, all tracklets in the pool are categorized into three states: tracked, lost, and removed. In the matching process, if the tracked and lost tracklets from the previous frame are successfully matched with the detected objects in the current frame, the tracked objects remain tracked and the lost objects are re-tracked until subsequent association. However, if there are unmatched tracklets or objects, we mark the unmatched tracklets as lost, while new tracklets are activated for the unmatched objects. Finally, we mark the tracklets as removed if they are lost right after the activation or have been lost for 30 frames in a row.

## 4. Experiments

### 4.1. Datasets

We train and evaluate our single-shot MOT system on the UA-DETRAC dataset [11]. All frames in the dataset are JPEG images with a resolution of 960 × 540 (width × height) and encompass extensive variations in object size, view, occlusion, and illumination. Our MOT system is trained on the UA-DETRAC-train set, which consists of 84 K frames in 60 sequences, 578 K bounding boxes, and 5.9 K IDs of vehicle objects. During training, we convert each bounding box, which is represented in the top-left corner, into the ground-truth box with respect to the object center. In addition, all vehicle types in the dataset are treated the same since our system does not classify vehicle objects into subclasses.

**Figure 5:**
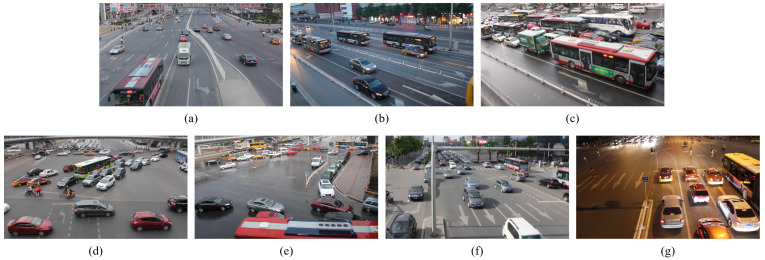
Sampled frames from each category of the UA-DETRAC-test subsets: (**a**) easy; (**b**) medium; (**c**) hard; (**d**) cloudy; (**e**) rainy; (**f**) sunny; (**g**) night.

We evaluate our MOT system on the UA-DETRAC-test set, which consists of 56 K frames in 40 sequences, 632 K bounding boxes, and 2.3 K IDs. To thoroughly analyze the performance of MOT, our system is further evaluated on the UA-DETRAC-test subsets that are categorized into three levels: easy, medium, and hard. The categorization is based on the average PR-MOTA score of the MOT systems built from previous studies [17,29,30,31,32,33,34,35,36,37]. Similarly, we also conduct experiments on the UA-DETRAC-test subsets that are classified into four categories: cloudy, rainy, sunny, and night, depending on the weather conditions and the intensity of illumination. Figure 5 shows the sampled frames from each category of the UA-DETRAC-test subsets.

### 4.2. Evaluation Metrics

We evaluate our single-shot MOT system based on the CLEAR MOT metrics [55] and UA-DETRAC metrics [11]. The CLEAR MOT metrics consist of several evaluation indicators that measure the MOT performance, including the multi-object tracking accuracy (MOTA), mostly tracked (MT), mostly lost (ML), ID switches (IDS), fragmentations (FM), false positives (FP), and false negatives (FN). Specifically, FP indicates the number of incorrect predictions for identifying background as objects, while FN is the opposite; that is, the number of incorrect predictions for recognizing objects as background. IDS stands for the number of ID changes of tracklets that are caused by mismatches, and FM is the number of tracklet disconnections induced by unmatches. The MOTA score represents the overall MOT accuracy of a system and is defined by Equation (11):

(11) $$\mathrm{MOTA}=\left(1-\frac{\sum_{t=1}^{T}\left({\mathrm{FP}}_{t}+{\mathrm{FN}}_{t}+{\mathrm{IDS}}_{t}\right)}{\sum_{t=1}^{T}{\mathrm{GT}}_{t}}\right)\cdot 100\;\left[\%\right]$$

where GT is the number of ground-truth objects and *T* is the total number of frames in the test set. MT and ML indicate the percentages of tracklets tracked by more than 80% and less than 20% of their lifetime on the ground-truth tracklets, respectively.

Based on the CLEAR MOT metrics, the UA-DETRAC metrics offer an extended evaluation protocol that thoroughly reflects the impact of the object detection performance on the overall MOT performance. The object detection performance is first evaluated by the precision-recall (PR) curve, which is generated by the precision and recall values measured with a confidence score threshold that continuously varies from zero to one. Here, the threshold of the IoU between the predicted and ground-truth bounding boxes is set to 0.7. Subsequently, the CLEAR MOT metrics are computed at different PR values by applying data association to the results of object detection, and each 3D PR-metric curve is plotted along the PR curve. The UA-DETRAC metrics, which include PR-MOTA, PR-MT, PR-ML, PR-IDS, PR-FM, PR-FP, and PR-FN, are finally computed using the line integral, which is the signed area between the corresponding PR-metric curve and the PR curve.

### 4.3. Implementation Details

Our single-shot MOT system is implemented in the experimental environment configured with Ubuntu 16.04 LTS OS, Intel Core I7-7700K CPU, two Nvidia GeForce GTX Titan X (Maxwell) GPUs, Pytorch 1.4.0, CUDA 10.1, and cuDNN 7.6.5. We use the EfficientDet-D0 [41] backbone network with parameters pre-trained on the MS COCO dataset [56] for initialization. The input frame is resized to a resolution of 1024 × 512 for a forward pass, and the corresponding resolution of the intermediate feature map for prediction heads is 256 × 128. We apply random rotation, scaling, and color jittering to the input frames for data augmentation. Our model is trained using the Adam optimizer [57] for 30 epochs with a batch size of 8. The learning rate is initialized to 10${}^{-4}$ and drops 0.1 times at the 20 epoch.

## 5. Results

### 5.1. Object Detection

**Figure 6:**
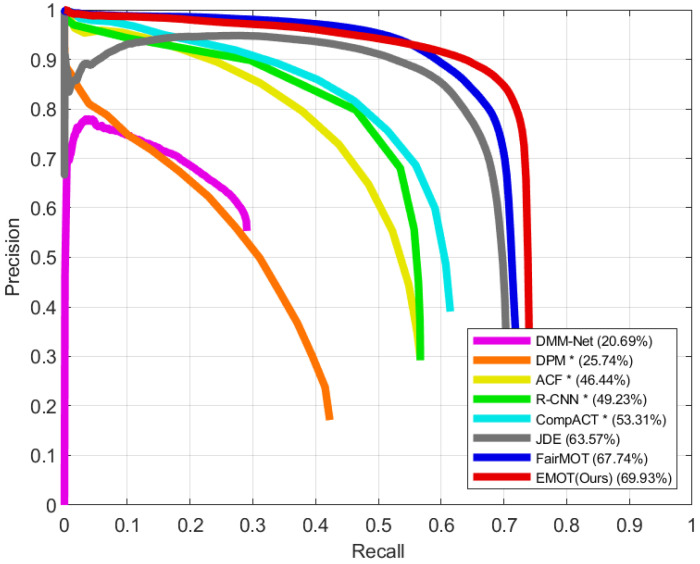
Precision-recall curves of our single-shot MOT system and different methods, which are evaluated on the UA-DETRAC-test set. The results of * were taken from Wen et al. [11].

We compare the object detection performance of our single-shot MOT system with the results of other detectors [17,29,30,31] and single-shot MOT systems [4,5,7] that are evaluated on the UA-DETRAC-test set [11]. Based on the PR curve, the AP score is computed by applying the PASCAL VOC Challenge [58] metric, which calculates the mean of precision values at 11 recall values that are equally placed from zero to one with an interval of 0.1. The IoU threshold is set to 0.7 for further use of the PR curve in the MOT evaluation using the UA-DETRAC metrics. The PR curves of our model and the different methods are compared in Figure 6. Our model, which is denoted by EMOT, shows the best performance for object detection, achieving an AP score of 69.93%. In this experiment, we do not specify the detector of the single-shot MOT systems, including EMOT, since they simultaneously perform object detection and embedding extraction.

The object detection results of our single-shot MOT system and different methods are shown in Table 2. EMOT shows the best performance, not only on the overall test set, but also for each test subset. We specifically compare EMOT to FairMOT [5], which shows the best performance among other methods. On the UA-DETRAC-test set, EMOT achieves a 2.2% higher AP score compared to FairMOT. In the UA-DETRAC-test subsets classified by difficulty, EMOT achieves the greatest improvement for the easy subset, achieving a 4.9% increased AP score. In contrast, the improvement is the least for the medium subset, which is 0.9%, whereas it is 2.2% for the hard subset. In the UA-DETRAC-test subsets classified by illumination, EMOT yields the most significant improvement for the rainy subset, achieving a 3.5% increase in the AP score. For the cloudy and sunny subsets, the increase in the AP score is 2.0% and 2.1%, respectively, while the night subset has only a 0.3% increased AP score.

### 5.2. Multi-Object Tracking

#### 5.2.1. CLEAR MOT Metrics

We compare our single-shot MOT system with other single-shot trackers [4,5,7] based on the MOT evaluation on the UA-DETRAC-test set [11] using the CLEAR MOT metrics [55]. The MOT results of our single-shot MOT system and different trackers are presented in Table 3. EMOT shows an overall greater MOT performance when compared to other methods, achieving a MOTA score of 68.5%. We observe that the improvement in the MOT performance is due to the enhancement of the object detection performance since both FP and FN scores reduce significantly. We also specifically compare EMOT to FairMOT [5], which shows the best MOT performance among other trackers. It is remarkable that EMOT achieves finer scores for both MT and ML, showing a 4.4% increase and 0.5% decrease, respectively. Only the IDS shows the results degraded, which increases by 52, whereas the FM decreases by 762 and gives better results. Notably, EMOT runs at an inference speed of 17.3 FPS and produces results comparable to JDE [4] and FairMOT, while showing markedly finer results for most of the metrics.

The MOT results of our single-shot MOT system and FairMOT, which are evaluated on the UA-DETRAC-test subsets using the CLEAR MOT metrics, are shown in Table 4. Based on a comparison of the MOTA scores, EMOT has a better MOT performance on all test subsets. In particular, EMOT presents the greatest improvement in the hard and night subsets with a 9.7% and 7.1% increase in the MOTA scores, respectively. Similar to the results for the overall test set, the metric scores for the test subsets are generally finer for EMOT, except for the IDS scores. Meanwhile, the MT and ML scores of EMOT are worse together, only in the night subset. We find out the reason from the FN score, which is also worse only in the same subset, since both MT and ML scores are heavily affected by the number of true positives.

#### 5.2.2. UA-DETRAC Metrics

We compare the MOT performance of our single-shot MOT system with the results of other trackers [17,20,29,30,31,32,33,34,35,36,37,38,59,60,61,62] that are evaluated on the UA-DETRAC-test set [11]. We note that the trackers being compared are built from the combinations of the detectors and association algorithms in a two-stage method. The MOT results of our single-shot MOT system and different trackers are presented in Table 5. EMOT achieves a PR-MOTA score of 24.5% and shows the best PR-ML and PR-FN scores. Based on a comparison of the PR-MOTA scores, EMOT outperforms most of the trackers, except for the state-of-the-art ones that use Mask R-CNN [20] as a detector. Compared to the Mask R-CNN + V-IOU [38] method, the PR-MOTA and PR-MT scores of EMOT decrease by 6.2% and 6.8%, respectively, owing to the inferior performance of object detection. However, the Mask R-CNN-based trackers present critical limitations in speed because they run at a low inference speed of 3.4 FPS, even without considering the runtime of the data association step, in the same experimental environment, as shown in Table 6. Furthermore, these trackers show degraded scores for the PR-ML when compared to most of the CompACT [31]-based trackers, while EMOT achieves the finest results with a PR-ML score of 9.3%. It is also noteworthy that the PR-FN score of EMOT is the lowest, indicating that EMOT produces the largest number of correct predictions for the object locations. Remarkably, EMOT presents a comparable PR-IDS score to the state-of-the-art methods by taking advantage of the appearance information of objects in matching IDs.

**Figure 7:**
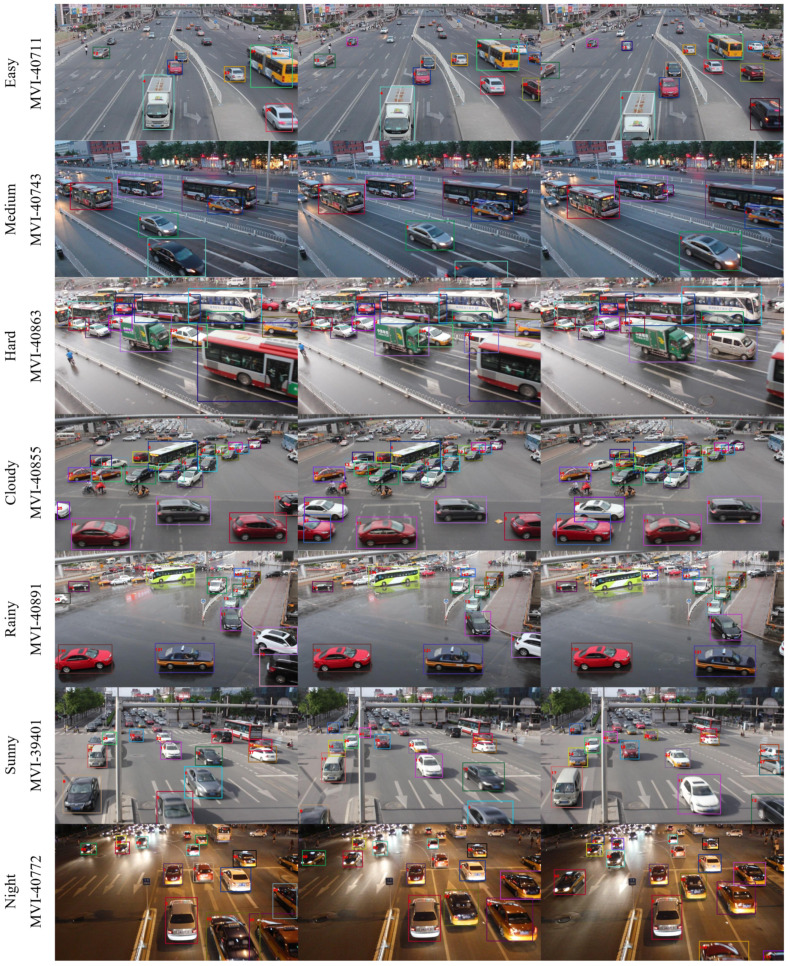
Qualitative results of our single-shot MOT system on the UA-DETRAC-test subsets. The frames are selected with a time interval of 20 frames.

The MOT results of our single-shot MOT system and FairMOT [5], which are evaluated on the UA-DETRAC-test set and subsets, are presented in Table 5 and Table 7, respectively. EMOT presents a better MOT performance than FairMOT on the evaluation using the UA-DETRAC metrics, achieving a 1.8% higher PR-MOTA score. In addition, all the results evaluated using the UA-DETRAC metrics are similar to those evaluated using the CLEAR MOT metrics [55]. Interestingly, a comparison of the scores for the UA-DETRAC metrics shows more consistent results than those for the CLEAR MOT metrics across the UA-DETRAC-test subsets. This tendency is particularly distinctive in the PR-IDS and PR-FP scores, where one surpasses the other in all test subsets. For an intuitive comprehension, we show the qualitative results of our single-shot MOT system on the UA-DETRAC-test subsets in Figure 7.

## 6. Conclusions

In this study, we propose a single-shot MOT system that achieves both high accuracy and efficiency by introducing an optimized backbone network and an efficient output-feature fusion. To extract multi-scale features that are more robust to scale variations, we utilize the EfficientDet-D0-based backbone network, which consists of the EfficientNet-B0 feature extractor and BiFPN fusion layers. In addition, we propose the FPN-based output-feature fusion using the deformable convolution in order to produce a single high-resolution feature map with richer representations from the transferred multi-scale features. By leveraging these finer features, our model performs well on both object detection and MOT. Specifically, our system presents remarkable results on vehicle tracking tasks, outperforming most of the state-of-the-art trackers on the UA-DETRAC dataset and running at a high inference speed. In the future, we expect to conduct systematic experiments to analyze and develop the structure and training for the re-identification network to reduce the ID switches in challenging environments with numerous occlusions. Furthermore, our future works will include extensions to various applications, such as multi-class and 3D MOT, and integrations with different tasks, such as simultaneous localization and mapping, and video compression.

## Figures and Tables

**Table 1:** Structure and specifications of the modified EfficientNet-B0.

Operation	Input Resolution (Width × Height)	# Output Channels	# Layers
Conv, k3×3	1024 × 512	32	1
MBConv1, k3×3	512 × 256	16	1
MBConv6, k3×3	512 × 256	24	2
MBConv6, k5×5	256 × 128	40	2
MBConv6, k3×3	128 × 64	80	3
MBConv6, k5×5	64 × 32	112	3
MBConv6, k5×5	64 × 32	192	4
MBConv6, k3×3	32 × 16	320	1

**Table 2:** Object detection results of our single-shot MOT system and different methods, which are evaluated on the UA-DETRAC-test set. Legends: D—detector; S—single-shot MOT system. The results of * were taken from Wen et al. [11].

Method	Type	Overall	Easy	Medium	Hard	Cloudy	Rainy	Sunny	Night
DPM * [29]	D	25.74	34.55	30.33	17.68	24.82	25.59	31.84	30.95
ACF * [30]		46.44	54.37	51.69	38.14	58.44	37.19	66.69	35.35
R-CNN * [17]		49.23	59.88	54.33	39.63	60.12	39.27	67.92	39.56
CompACT * [31]		53.31	64.94	58.80	43.22	63.30	44.28	71.25	46.47
DMM-Net [7]	S	20.69	31.21	23.60	14.03	26.10	14.56	36.89	15.01
JDE [4]		63.57	79.84	70.46	49.88	76.02	50.42	73.00	58.92
FairMOT [5]		67.74	81.33	73.32	56.78	77.21	55.46	75.44	69.05
EMOT (Ours)		69.93	86.19	74.26	59.00	79.20	59.00	77.55	69.32

**Table 3:** MOT results of our single-shot MOT system and different trackers, which are evaluated on the UA-DETRAC-test set using the CLEAR MOT metrics. All inference speeds are measured with a single Nvidia Geforce RTX 2080 SUPER GPU. The ↑ indicates that a higher score is better, while ↓ indicates that a lower score is better.

Method	MOTA ↑	MT ↑	ML ↓	IDS ↓	FM ↓	FP ↓	FN ↓	FPS ↑
DMM-Net [7]	20.3	19.9	30.3	498	1428	104,142	399,586	101.2
JDE [4]	55.1	68.4	6.5	2169	4224	128,069	153,609	17.6
FairMOT [5]	63.4	64.0	7.8	784	4443	71,231	159,523	18.9
EMOT (Ours)	68.5	68.4	7.3	836	3681	50,754	147,383	17.3

**Table 4:** MOT results of our single-shot MOT system and FairMOT, which are evaluated on the UA-DETRAC-test subsets using the CLEAR MOT metrics. The scores on the left side indicate the results of FairMOT, while those on the right side indicate the results of our single-shot MOT system.

Subset	MOTA ↑	MT ↑	ML ↓	IDS ↓	FM ↓	FP ↓	FN ↓
Easy	82.8/84.9	81.8/86.0	1.2/1.4	62/121	498/379	10,159/9276	11,304/9468
Medium	65.3/69.6	61.0/66.8	8.7/8.0	445/421	2590/2056	34,081/27,169	89,466/80,934
Hard	42.5/52.2	46.8/47.9	14.5/13.5	277/294	1355/1246	26,991/14,309	58,753/56,981
Cloudy	77.0/79.5	71.1/80.0	3.5/3.5	147/141	1216/738	12,749/12,272	32,191/27,800
Rainy	50.9/57.4	46.8/54.2	16.4/14.5	264/270	1553/1413	24,689/17,300	76,002/70,055
Sunny	68.7/73.1	77.0/78.1	3.7/2.4	65/95	488/343	8913/8253	14,217/11,605
Night	60.3/67.4	64.2/63.9	6.6/7.5	308/330	1186/1187	24,880/12,929	37,113/37,923

**Table 5:** MOT results of our single-shot MOT system and different trackers, which are evaluated on the UA-DETRAC-test set. The results of * were taken from Wen et al. [11] and the rest of the results were taken from their own papers, except for FairMOT.

Detection	Association	PR-MOTA ↑	PR-MT ↑	PR-ML ↓	PR-IDS ↓	PR-FM ↓	PR-FP ↓	PR-FN ↓
DPM [29]	GOG * [32]	5.5	4.1	27.7	1873.9	1988.5	38,957.6	230,126.6
	CEM * [33]	3.3	1.3	37.8	265.0	317.1	13,888.7	270,718.5
	DCT * [34]	2.7	0.5	42.7	72.2	68.8	7785.8	280,762.2
ACF [30]	GOG * [32]	10.8	12.2	22.3	3950.8	3987.3	45,201.5	197,094.2
	DCT * [34]	7.9	4.8	34.4	108.1	101.4	13,059.7	251,166.4
	H${}^{2}$T * [36]	8.2	13.1	21.3	1122.8	1445.8	71,567.4	189,649.1
R-CNN [17]	DCT * [34]	11.7	10.1	22.8	758.7	742.9	36,561.2	210,855.6
	H${}^{2}$T * [36]	11.1	14.6	19.8	1481.9	1820.8	66,137.2	184,358.2
	CMOT * [37]	11.0	15.7	19.0	506.2	2551.1	74,253.6	177,532.6
CompACT [31]	GOG * [32]	14.2	13.9	19.9	3334.6	3172.4	32,092.9	180,183.8
	CEM * [33]	5.1	3.0	35.3	267.9	352.3	12,341.2	260,390.4
	DCT * [34]	10.8	6.7	29.3	141.4	132.4	13,226.1	223,578.8
	IHTLS * [35]	11.1	13.8	19.9	953.6	3556.9	53,922.3	180,422.3
	H${}^{2}$T * [36]	12.4	14.8	19.4	852.2	1117.2	51,765.7	173,899.8
	CMOT * [37]	12.6	16.1	18.6	285.3	1516.8	57,885.9	167,110.8
	FAMNet [59]	19.8	17.1	18.2	617.4	970.2	14,988.6	164,432.6
EB [60]	IOU [61]	19.4	17.7	18.4	2311.3	2445.9	14,796.5	171,806.8
	DAN [62]	20.2	14.5	18.1	518.2	-	9747.8	135,978.1
Mask R-CNN [20]	IOU [61]	30.7	30.3	21.5	668.0	733.6	17,370.3	179,505.9
	V-IOU [38]	30.7	32.0	22.6	162.6	286.2	18,046.2	179,191.2
FairMOT [5]		22.7	23.7	10.0	347.1	2993.6	49,385.4	123,124.5
EMOT (Ours)		24.5	25.2	9.3	379.0	2957.3	43,940.6	116,860.7

**Table 6:** Inference speeds of our single-shot MOT system and different methods. Legends: D-object detection; E-embedding extraction. The results of * were taken from Wen et al. [11].

Method	Tasks	Environment (CPU & GPU)	FPS
DPM * [29]	D	4 Intel Core i7-6600U (2.60 GHz)	0.17
		-	
ACF * [30]		2 Intel Xeon E5-2470v2 (2.40 GHz)	0.67
		-	
R-CNN * [17]		2 Intel Xeon E5-2470v2 (2.40 GHz)	0.10
		Tesla K40	
CompACT * [31]		2 Intel Xeon E5-2470v2 (2.40 GHz)	0.22
		Tesla K40	
EB * [60]		4 Intel Core i7-4770 (3.40 GHz)	11.0
		Titan X	
Mask R-CNN [20]		Intel Core i7-10700K (3.80 GHz)	3.4
		Nvidia Geforce RTX 2080 SUPER	
FairMOT [5]	D + E	Intel Core i7-10700K (3.80 GHz)	18.9
		Nvidia Geforce RTX 2080 SUPER	
EMOT		Intel Core i7-10700K (3.80 GHz)	17.3
		Nvidia Geforce RTX 2080 SUPER	

**Table 7:** MOT results of our single-shot MOT system and FairMOT, which are evaluated on the UA-DETRAC-test subsets.

Subset	PR-MOTA ↑	PR-MT ↑	PR-ML ↓	PR-IDS ↓	PR-FM ↓	PR-FP ↓	PR-FN ↓
Easy	29.7/30.8	30.8/32.3	5.2 /5.2	42.7 /56.8	644.5 /648.3	10,371.5/10,022.7	15,027.4/14,017.6
Medium	24.3/25.8	22.4/24.3	10.7/9.9	189.0/199.0	1652.4/1613.1	23,031.3/21,497.6	68,472.9/64,824.3
Hard	12.8/16.2	17.1/17.6	15.1/13.4	115.4/123.1	696.7 /695.8	15,982.6/12,420.3	39,624.1/38,018.9
Cloudy	29.4/30.9	25.4/28.7	7.8 /7.6	70.3 /76.0	973.0 /925.5	8485.1 /8481.9	31,822.1/28,943.4
Rainy	18.5/20.9	17.4/19.6	16.0/14.0	111.0/122.3	866.0 /849.2	13,710.9/11,493.5	50,931.3/48,182.2
Sunny	20.5/22.8	28.5/29.5	5.8 /5.4	30.6 /41.5	370.6 /390.4	9823.6 /9354.7	11,964.8/10,777.2
Night	20.7/22.1	24.5/23.9	9.6 /9.3	135.2/139.2	783.9 /792.2	17,365.8/14,610.5	28,406.3/28,958.0
